# BDNF levels in serum and CSF are associated with clinicoradiological characteristics of aggressive disease in MS patients

**DOI:** 10.1007/s00415-024-12875-3

**Published:** 2025-01-15

**Authors:** Michelle Maiworm, Kimberly Koerbel, Victoria Anschütz, Jasmin Jakob, Martin A. Schaller-Paule, Jan Hendrik Schäfer, Lucie Friedauer, Katharina J. Wenger, Maya C. Hoelter, Falk Steffen, Stefan Bittner, Christian Foerch, Yavor Yalachkov

**Affiliations:** 1 Department of Neurology, University Hospital Frankfurt, Frankfurt Am Main, Germany; 2 Department of Neurology, Focus Program Translational Neuroscience (FTN) and Immunotherapy (FZI), Rhine Main Neuroscience Network (Rmn2), University Medical Center of the Johannes Gutenberg University Mainz, Mainz, Germany; 3 Practice for Neurology and Psychiatry Eltville, Eltville Am Rhein, Germany; 4 Institute of Neuroradiology, University Hospital Frankfurt, Frankfurt Am Main, Germany; 5 Department of Radiology, Sankt Katharinen Hospital, Frankfurt Am Main, Germany; 6 Department of Neurology, RKH Klinikum Ludwigsburg, Ludwigsburg, Germany

**Keywords:** Brain-derived neurotrophic factor, Aggressive MS, Multiple sclerosis, NfL, GFAP

## Abstract

**Background:**

BDNF has increasingly gained attention as a key molecule controlling remyelination with a prominent role in neuroplasticity and neuroprotection. Still, it remains unclear how BDNF relates to clinicoradiological characteristics particularly at the early stage of the disease where precise prognosis for the further MS course is crucial.

**Methods:**

BDNF, NfL and GFAP concentrations in serum and CSF were assessed in 106 treatment na﻿ïve patients with MS (pwMS) as well as 73 patients with other inflammatory/non-inflammatory neurological or somatoform disorders using a single molecule array HD-1 analyser. PwMS were evaluated for highly active profiles by applying the aggressive disease course criteria proposed by ECTRIMS. Serum/CSF values were logarithmically transformed and compared across groups using one-way ANOVA, while correlations were calculated using Pearson’s correlations. ROC analysis and AUC comparisons for diagnostic performance of the three biomarkers were computed in an explorative analysis.

**Results:**

Serum BDNF (sBDNF) concentrations were higher in treatment naïve pwMS with disease onset after the age of 40 years (*p* = 0.029), in pwMS with ≥2 gadolinium-enhancing lesions (*p* = 0.009) and with motor, cerebellar, cognitive or sphincter symptoms at onset (*p* = 0.036). BDNF correlated positively with NfL (*r* = 0.198, *p* = 0.014) and GFAP (*r* = 0.253, *p* = 0.002) in serum, but not in CSF. Neurological patients with an acute inflammatory relapse showed significantly higher sBDNF levels (*p* = 0.03) compared to somatoform controls, while patients without acute relapse did not differ from somatoform controls (*p* = 0.4). Better diagnostic performance was found for sBDNF than sNfL and sGFAP in differentiating between patients with vs. without 2 or more gadolinium-enhancing lesions (*p* < 0.05) and for sBDNF as compared to sNfL for separating patients with disease onset after vs. before age of 40 years.

**Conclusion:**

In pwMS, BDNF serum levels differ depending on disease-related characteristics, suggesting that not only inflammatory activity but also remyelination capacities may vary with disease severity. BDNF is increased when other biomarkers of neuroaxonal damage and neurodegeneration, such as NfL and GFAP, are elevated, possibly as a compensatory mechanism, and reflect possibly further pathophysiological aspects in MS beyond NfL and GFAP, probably including an apoptotic role for BDNF in neuroinflammation.

## Introduction

Recent research in the field of multiple sclerosis (MS) has focused on the investigation of factors driving demyelination and neurodegeneration. However, histopathological studies have shown that in patients with MS (pwMS), especially with pronounced neurodegeneration, remyelinating processes exist besides neuroinflammation [1, 2]. Thus, lack of repair mechanisms and insufficient remyelination might result in accumulating neuronal injury and long-term disability [3]. Especially with highly effective therapies designed to diminish inflammation and delay neurodegeneration, approaches aiming to reverse preexisting injury are becoming increasingly important.

In this context, a member of the neurotrophin family, the brain-derived neurotrophic factor (BDNF) has received particular attention as it is thought to play a central role in remyelination [4–7], as proliferation as well as maturation of oligodendrocyte progenitor cell (OPCs) can be stimulated by BDNF via its high affinity receptor TrkB [8–10], with supporting evidence from animal models [1]. BDNF also appears to play a central role in neuroplasticity and learning as well as memory by causing hippocampal long-term potentiation and neurogenesis of hippocampus and gyrus dentatus [11].

Though BDNF has been already studied in several neurological and psychiatric diseases as a promising marker of disease activity with remyelination potential, it remains unclear whether in patients with inflammatory neurological diseases and high disease activity and thus accumulating disability, remyelinating effects are insufficient due to failure of this processes or whether they are even upregulated as a compensatory mechanism but not sufficient to prevent long-term disability. In MS, lower BDNF concentrations—mostly in serum, but also in CSF—were found compared to healthy controls in most studies [12–16] though some studies showed no difference [12, 17–19] or even higher BDNF levels in MS patients [12, 14, 16, 20–23]. During and after acute relapses, serum BDNF levels have been found increased or unchanged [12–14, 18, 21, 24].

Still, results from studies assessing the association between BDNF concentrations and clinicoradiological characteristics remained inconclusive. Several clinicoradiological characteristics as increasing EDSS and reaching of EDSS milestones, two or more relapses per year as well as severe relapses with poor recovery, older age at disease onset and MRI characteristics as high lesion load or presence of gadolinium-enhancing lesions were considered as hallmarks of an aggressive disease course with high inflammatory disease activity by the European Committee for Treatment and Research in Multiple Sclerosis (ECTRIMS) consensus group [25].

On a pathophysiological level, increasing BDNF level in presence of inflammatory disease activity would be likely as damaged astrocytes as well as microglia and macrophages are able to express and secrete neurotrophic factors as BDNF [26]. Accordingly, higher BDNF concentrations would be expected in patients with those clinicoradiological characteristics of an aggressive disease course. Still, some studies failed to find an association between disease severity and BDNF concentration [27], while an association of higher BDNF concentrations with EDSS improvement after 12 months was found in another study [28].

Based on the effects of BDNF on memory performance demonstrated in animal models, a relationship between BDNF concentration and cognitive deficits has also been hypothesized in the context of MS [29, 30] as well as other neurological diseases. In this context, it has been assumed that the pathological changes in pwMS leading to cognitive impairment are compensated through hippocampal hyperactivation, potentially related to higher BDNF levels [31–35], to preserve episodic memory [31, 36, 37].

Regarding MRI characteristics, a negative correlation between BDNF and T2- [38] and T1-lesion volume [39] as marker for inflammatory as well as neurodegenerative axonal damage has been reported. However, some studies did not find a correlation of BDNF concentration and T1-lesion volume [38]. Furthermore, higher BDNF levels are associated with microscopic damage in the normal appearing white matter, leading to the assumption that secretion of BDNF is increased in the early formation of inflammatory lesions or diffuse inflammatory infiltrates without correlates in conventional MRI [40]. Still, no relationship between gadolinium-enhancing lesions as manifestation of acute inflammation and BDNF was found [38].

It therefore remains unclear how BDNF relates to clinical, laboratory and MRI characteristics as hallmarks of disease activity in MS and other neurological, especially neuroinflammatory diseases. In the context of MS, this is particularly important for early-stage patients where a precise prognosis of the future disease course is crucial.

The aim of this study was therefore to explore the association of serum and CSF BDNF (sBDNF/cBDNF) concentrations with relevant clinical, laboratory and MR-imaging parameters considered as aggressive disease course criteria as proposed by ECTRIMS [25] in treatment naïve pwMS as well as patients with other inflammatory neurological diseases (IND).

## Methods

### Study population

Patients with chronic inflammatory CNS diseases, such as multiple sclerosis, neuromyelitis optica spectrum disorders (NMOSD), MOG-antibody-associated diseases (MOGAD), sarcoidosis of the CNS without disease-modifying therapy as well as other non-inflammatory neurological diseases (NIND) (e.g., neurodegenerative diseases, polyneuropathies, CNS tumors) and controls with a somatoform disease, were recruited between October 2017 and December 2020 at the Department of Neurology at the University Hospital Frankfurt. In all neuroinflammatory diseases, relapses were defined as a (sub-)acute onset of new neurological symptoms or relevant worsening of existing symptoms after ruling out other possible causes. Somatoform disease controls were patients presenting with neurological symptoms as paresthesia or palsy whose full diagnostic work-up did not reveal an underlying organic disease. Only patients who had a clinically indicated lumbar puncture were included in the study. All subjects underwent a neurological examination, assessing physical disability using the Kurtzke Expanded Disability Status Scale (EDSS) [41]. MRI of the brain and spinal cord, lumbar puncture and blood analysis were performed during diagnostic work-up. Serum and CSF samples for biomarker analysis were collected during clinically scheduled sample collection and before any corticosteroid therapy. Patients with MS were classified according to the clinical, laboratory and imaging characteristics of aggressive disease course as proposed by ECTRIMS [25].

Exclusion criteria were age below 18 years as well as patients unable to give written informed consent to participate in the study.

The study was performed in accordance with The Code of Ethics of the World Medical Association (Declaration of Helsinki) for experiments involving humans and was approved by the local ethics committee at the University Hospital Frankfurt. Written informed consent was obtained from all subjects before enrollment.

### Serum and CSF measurements

Blood (S-Monovette, 4.7 mL, Sarstedt, containing coagulation activating agent) and CSF samples (Greiner PS, 14 ml) were centrifuged at 4000 runs per minute (rpm) for 10 min at 4 degrees Celsius. Afterward, serum was pipetted and frozen at −20 degrees Celsius within 60 min after collection. Within 4 weeks at the latest, the samples were frozen to −80 degrees Celsius. For quantification of NfL, GFAP and BDNF concentrations, the frozen blood and CSF samples were sent to the Department of Neurology at the University of Mainz. Quantification of biomarkers was performed using the Single Molecule Array (SIMOA) HD-1 analyzer (Quanterix).

Furthermore, in CSF, leucocytes per mm^3^ were counted manually, CSF/serum albumin quotient, intrathecal immunoglobulin G synthesis as well as oligoclonal bands were assessed. Assessment of blood–brain barrier disruption and CSF/serum albumin quotient was interpreted as suggested by Reiber et al. [42]. In patients suffering an acute relapse, blood and CSF samples were assessed within 6 weeks after relapse onset.

### Magnetic resonance imaging

MRI of the brain and spinal cord was performed during clinical routine measurements. Lesion count was assessed on 2D T2-weighted (T2w) sequences and, if available for brain imaging, reconciled on 2D or 3D fluid-attenuated inversion recovery (FLAIR) or 3D double inversion recovery (DIR) sequences. Presence and number were confirmed by two experienced clinical neuroradiologists in a consensus process (M.H. >15 years and K.W. >8 years of experience in neuroradiological imaging). The existence of infratentorial and spinal cord lesions was recorded. The presence of gadolinium-enhancing (GAD+) lesions was evaluated on gadolinium-enhanced T1-weighted sequences [43].

### Statistical analysis

For the descriptive statistics, mean values and standard deviations as well as median values and interquartile range (IQR) were calculated.

BDNF, NfL and GFAP concentrations were logarithmically transformed. Correlations between BDNF with NfL and GFAP were calculated using Pearson’s correlations.

Group comparisons were calculated via one-way analysis of variance (ANOVA) with BDNF serum and CSF levels as dependent variable and the clinicoradiological characteristics for aggressive disease course as proposed by the ECTRIMS consensus group [25] as independent variables.

To further explore whether serum biomarker levels perform differently with regard to differentiating between patients having and those without specific hallmarks of aggressive MS course, receiver operator characteristic (ROC) curve analysis was performed for each biomarker, yielding specific area under the curve (AUC) values, which were tested for significance. For biomarkers differentiating between patients in the previous group comparison analysis (e.g., between patients with 2 or more gadolinium-enhancing lesions on MRI and those with less lesions), AUC values and thus diagnostic performance were compared for each biomarker pair by employing z-statistics. All significance levels were set at <0.05.

## Results

### Study population

The study included 106 pwMS (22 with clinically isolated syndrome, 73 with relapsing–remitting and 11 with primary progressive MS according to the 2017 revised McDonald criteria [44]) without disease-modifying therapy as well as 51 patients with other neurological diseases (e.g., MOGAD, NMOSD, neurosarcoidosis, polyneuropathies, neurodegenerative diseases, CNS tumors). Furthermore, 22 patients with somatoform diseases were included. Table 1 outlines the demographic and clinical characteristics of all included subjects in detail.

**Table 1:** Demographic and clinical characteristics of MS patients, patients with other neurological diseases as well as somatoform controls

	MS patients (*N* = 106)	Other neurological diseases (*N* = 51)	Somatoform controls (*N* = 22)
Sex female *(%)*	77 *(72.6)*	27 *(52.9)*	17 *(72.3)*
Age at time of study enrolment [in years ± SD]	35.29 ± 10.82 y (range 19–69 y)	41.28 ± 15.11 y (range 20–74 y)	35.36 ± 10.05 y (range 19–55 y)
BMI [kg/m^2^ ± SD]	24.98 ± 5.09 (range 16.7–46.3)	25.34 ± 5.35 (range 17.6–44.1)	23.75 ± 8.91 (range 15.8–48.3)
Disease type			
CIS	22 *(20.8%)*		
RRMS	73 *(68.8%)*		
PPMS	11 *(10.4%)*		
Acute relapse yes *(%)*/no *(%)*	84 *(79.3)*/22 *(20.8)*	22 *(43.1)*/29 *(56.9)*	
EDSS (±SD)			
with relapse	2,07 ± 1,07 (range 0–4.5)	1.81 ± 1.05 (range 0–4)	
without relapse	1.75 ± 1.73 (range 0–5)	0.07 ± 0.27 (range 0–1)	
Number of relapses 12 months prior to Study enrolment (±SD)	1.03 ± 0.72 (range 0–6)	0.56 ± 0.68 (range 0–3)	

### Matching biomarker concentrations to clinical, laboratory and MRI characteristics in treatment naïve patients with MS

**Fig. 1:**
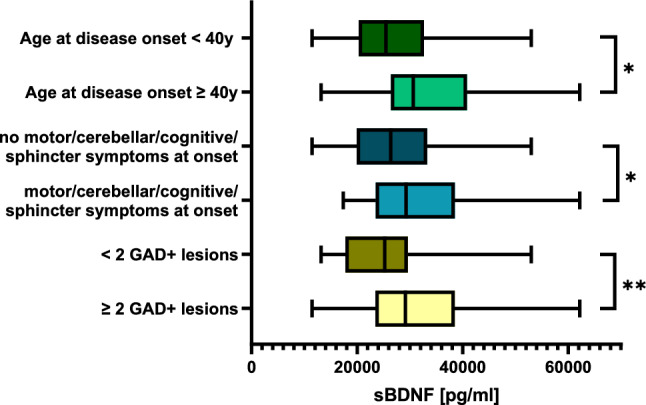
Association of BDNF concentration in serum to different clinicoradiological characteristics of an aggressive disease course. sBDNF concentrations were significantly higher in treatment naïve pwMS with disease onset after the age of 40 years (*p* = 0.029) as well as pwMS with motor, cerebellar, cognitive or sphincter symptoms at onset (*p* = 0.036) and with ≥ 2 Gadolinium-enhancing lesions (*p* = 0.009). No differences were found regarding cBDNF concentrations

sBDNF concentrations were significantly higher in treatment naïve pwMS with disease onset after the age of 40 years (*p* = 0.029, mean ± SD 27039.61 ± 9508.80 pg/ml vs 33032.93 ± 11852.98 pg/ml, Fig. 1). Moreover, patients with motor, cerebellar, cognitive or sphincter symptoms at onset showed higher sBDNF concentrations than patients without these symptoms at onset (*p* = 0.036, mean ± SD 32413.41 ± 10528.35 pg/ml vs 27318.37 ± 10179.79 pg/ml, Fig. 1).

sBDNF did not differentiate between patients exhibiting or not exhibiting other clinical characteristics considered as risk factors for an aggressive disease course [25].

Regarding MRI characteristics, patients with ≥2 gadolinium-enhancing lesions showed significantly higher sBDNF concentrations (*p* = 0.009, mean ± SD 31051.16 ± 10323.09 pg/ml vs 25429.45 ± 9391.19 pg/ml, Fig. 1). The lesion count as well as the presence of spinal cord lesions or infratentorial lesions did not influence sBDNF concentrations. Similarly, laboratory parameters, such as intrathecal IgG synthesis, CSF-specific oligoclonal bands as well as blood–brain barrier dysfunction, EDSS, severe relapses, poor recovery (defined as residual symptoms relevant to daily life despite exhaustion of relapse therapy), or lesion count, did not show an association with BDNF concentration. No significant associations of clinicoradiological characteristics with BDNF concentration in CSF were found.

Table 2 provides an overview of the BDNF concentrations for the different clinicoradiological and laboratory characteristics.

**Table 2:** BDNF concentrations in serum and CSF for different clinical, laboratory and MRI characteristics

Clinical characteristics	N (%)	sBDNF [pg/ml] mean ± SD	*p* value	cBDNF [pg/ml] mean ± SD	*p* value
Sex					
Female	77 (72.6)	28726 ± 10401	n.s	0.1523 ± 0.3635	n.s
Male	29 (37.4)	27749 ± 9993		0.3964 ± 1.447	
BMI					
<25 kg/m^2^	56 (58.3)	26690 ± 9220	n.s	0.1288 ± 0.3241	n.s
≥25 kg/m^2^	40 (41.7)	30651 ± 10694		0.3568 ± 1.252	
Acute relapse					
Yes	84 (79.3)	28952 ± 10799	n.s	0.2245 ± 0.8632	n.s
No	22 (20.8)	25754 ± 10889		0.1938 ± 0.5987	
Severe relapses					
Yes	36 (37.9)	28520 ± 8456	n.s	0.0887 ± 0.3140	n.s
No	57 (62.1)	28386 ± 11528		0.2612 ± 1.043	
Poor recovery					
Yes	21 (22.3)	30082 ± 11232	n.s	0.0384 ± 0.2061	n.s
No	73 (77.7)	27899 ± 9991		0.2610 ± 0.9602	
Age at symptom onset					
Age <40 y at onset	72 (76.6)	27040 ± 9509	*0.029*	0.2306 ± 0.9223	n.s
Age ≥40 y at onset	22 (23.4)	33033 ± 11853		0.2617 ± 0.6386	
EDSS					
EDSS ≤ 3 in 1st year	73 (78.5)	28127 ± 10700	n.s	0.2460 ± 0.9476	n.s
EDSS > 3 in 1st year	20 (21.5)	28698 ± 8709		0.0475 ± 0.2360	
Motor symptoms at onset					
Yes	15 (16.0)	30134 ± 8944	n.s	0.2402 ± 0.5233	n.s
No	79 (84.0)	28088 ± 10595		0.2376 ± 0.9136	
Motor, cerebellar, cognitive or sphincter symptoms at onset					
Yes	21 (22.6)	32413 ± 10528	0.036	0.0606 ± 0.2635	n.s
No	72 (77.4)	27442 ± 10160		0.2320 ± 0.9478	
Pyramidal signs in the first year					
Yes	21 (22.6)	30045 ± 8181	n.s	0.04832 ± 0.2314	n.s
No	72 (77.4)	27786 ± 10932		0.2484 ± 0.9557	
MRI characteristics					
Number of cerebral lesions			n.s		n.s
0 lesions	11 (12.1)	29624 ± 8802		0.1529 ± 0.3736	
1–3 lesions	15 (16.5)	28075 ± 10238		0.2187 ± 0.5139	
4–9 lesions	26 (28.6)	30190 ± 12369		0.0666 ± 0.9675	
10–20 lesions	17 (18.7)	24229 ± 9764		0.2893 ± 0.7150	
>20 lesions	22 (24.1)	30702 ± 8433		0.5125 ± 1.5700	
Infratentorial lesions					
Yes	37 (40.2)	28378 ± 8963	n.s	0.0785 ± 0.2749	n.s
No	55 (59.8)	29069 ± 11,290		0.3022 ± 1.0980	
Spinal cord lesions					
Yes	40 (74.1)	27864 ± 8967	n.s	0.0822 ± 0.2863	n.s
No	14 (25.9)	32172 ± 3084		0.0916 ± 0.1224	
Gadolinium-enhancing lesions					
≥2 GAD + lesions	48 (54.5)	31051 ± 10323	0.009	0.2006 ± 0.8698	n.s
<2 GAD + lesions	40 (45.5)	26423 ± 11058		0.1298 ± 0.3640	
Laboratory characteristics					
IgG synthesis in CSF					
Yes	64 (60.4)	28345 ± 10711	n.s	0.1120 ± 0.3242	n.s
No	42 (39.6)	28684 ± 9683		0.3208 ± 1.209	
CSF-specific OCBs					
Yes	87 (91.6)	28110 ± 10352	n.s	0.2485 ± 0.8406	n.s
No	8 (8.4)	32940 ± 8395		0.0403 ± 0.3839	
Blood–brain barrier leak					
Yes	20 (22.7)	29223 ± 11814	n.s	0.1948 ± 0.6241	n.s
No	68 (77.3)	28381 ± 9990		0.2236 ± 0.8521	

However, we did not find any significant differences in sNFL or sGFAP levels between patients with disease onset before vs. after the age of 40 yeas (log-sNfL: *p* = 0.703, 1.111 ± 0.285 vs. 1.086 ± 0.210; log-sGFAP: 1.936 ± 0.209 vs. 1.955 ± 0.142), patients without motor, cerebellar, cognitive or sphincter symptoms at onset vs. those with these symptoms at onset (log-sNfL: *p* = 0.260, 1.087 ± 0.268 vs. 1.164 ± 0.274; log-sGFAP: *p* = 0.392, 1.931 ± 0.206 vs. 1.973 ± 0.154) or patients with less than two gadolinium-enhancing lesions vs. those with at least two gadolinium-enhancing lesions at disease onset (log-sNfL: *p* = 0.615, 1.119 ± 0.270 vs. 1.090 ± 0.265; log-sGFAP; *p* = 0.792, 1.952 ± 0.209 vs. 1.941 ± 0.187).

### ROC analysis and AUC comparisons between biomarkers

**Fig. 2:**
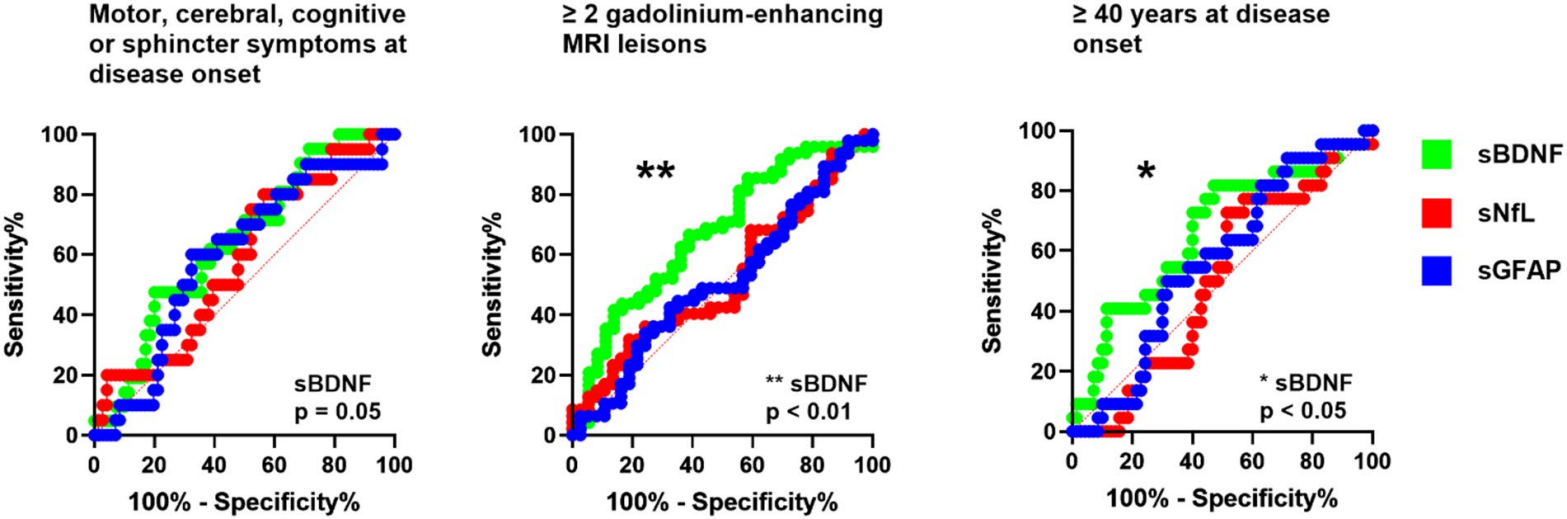
Receiver operator characteristic (ROC) curve analysis of the biomarkers for differentiation between MS patients with different clinicoradiological characteristics. sBDNF was borderline significant (*p* = 0.05) for differentiating between patients with motor, cerebellar, cognitive or sphincter symptoms and those without these symptoms at onset compared to sNfL and sGFAP, while it was significant for differentiating patients with two or more and those with less than two gadolinium-enhancing lesions as well as those at least 40 years old vs. those younger than 40 years at disease onset (both *p* = 0.01)

sBDNF concentration yielded significant AUC values when differentiating between patients with two or more and those with less than two gadolinium-enhancing lesions as well as those at least 40 years old vs. those younger than 40 years at disease onset (both *p* = 0.01). For differentiating between patients with motor, cerebellar, cognitive or sphincter symptoms and those without such symptoms at disease onset, sBDNF was borderline significant (*p* = 0.05). Neither sNfL nor sGFAP reached significance in the ROC analysis of those three variables (*p* > 0.05). The results of the ROC analysis are illustrated in Fig. 2 and in Table 3.

**Table 3:** ROC analysis and AUC comparisons between biomarkers

ROC analysis															
	Motor, cerebellar, cognitive or sphincter symptoms at onset					≥2 GAD + lesions					Age ≥ 40 y at onset				
	AUC	Std. error	95% confidence interval		*p* value	AUC	Std. error	95% confidence interval		*p* value	AUC	Std. error	95% confidence interval		*p* value
			lower	upper				lower	upper				lower	upper	
sBDNF	0.63	0.07	0.48	0.74	0.05	0.67	0.06	0.53	0.77	0.01	0.67	0.07	0.51	0.78	0.01
sNfL	0.58	0.07	0.43	0.70	n.s	0.47	0.06	0.34	0.59	n.s	0.50	0.07	0.36	0.62	n.s
sGFAP	0.60	0.07	0.44	0.72	n.s	0.48	0.07	0.35	0.60	n.s	0.56	0.07	0.42	0.68	n.s

AUC comparisons															
	*z*-value			*p* value		*z*-value			*p* value		*z*-value			*p* value	
sBDNF vs. sNfL	0.51			n.s		2.41			0.02		2.09			0.04	
sBDNF vs. sGFAP	0.36			n.s		2.09			0.04		1.07			n.s	
sGFAP vs. sNfL	−0.18			n.s		−0.18			n.s		−0.92			n.s	

When comparing directly the AUC between each pair of biomarkers, sBDNF exhibited significantly larger AUC than sNfL (*p* = 0.02) and sGFAP (*p* = 0.04) when differentiating between patients with two or more and those with less than two gadolinium-enhancing lesions. Additionally, sBDNF demonstrated larger AUC than sNfL (*p* = 0.04) when comparing patients with disease onset after vs. before the age of 40 years. Table 3 illustrates the results of the AUC comparisons.

### Biomarker concentrations in neurological diseases

**Fig. 3:**
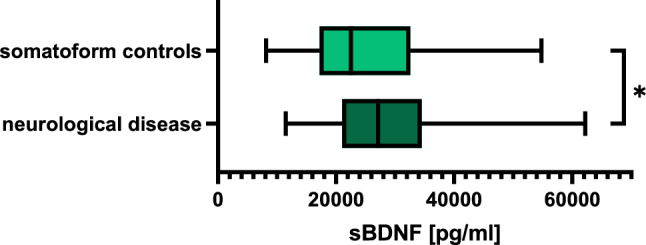
Boxplots showing BDNF concentrations in serum for somatoform controls compared to patients with neurological disease. Higher sBDNF levels were found in patients with a neurological disease compared to somatoform controls (*p* = 0.044)

In general, patients with a neurological disease showed significantly higher sBDNF levels than somatoform controls (*p* = 0.044, Fig. 3), while no difference was found for cBDNF levels.

**Fig. 4:**
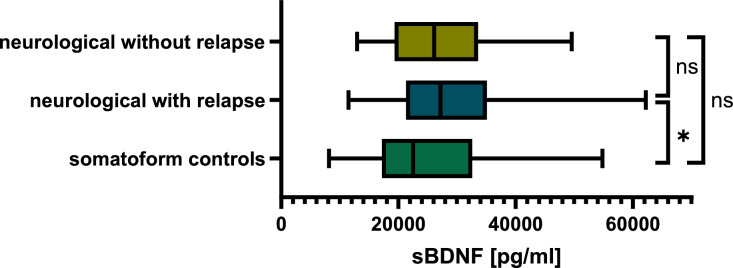
Boxplots showing BDNF concentrations in serum for somatoform controls compared to patients with neuroinflammatory disease without and with relapse. Neurological patients with an acute inflammatory relapse showed significantly higher sBDNF levels than somatoform controls (*p* = 0.03), while no significant differences were found between neurological patients without inflammatory relapse and somatoform controls (*p* = 0.4)

In 61% of the neurological patients, serum and CSF samples were collected during an acute relapse. In a subgroup analysis comparing BDNF concentrations only in IND with an acute inflammatory relapse (e.g., pwMS, MOGAD, NMOSD, neurosarcoidosis, etc.), sBDNF was still significantly higher (*p* = 0.03, Fig. 4), while neurological patients without acute relapse did not differ regarding their sBDNF levels compared to somatoform controls (*p* = 0.4, Fig. 4). Again, no significant difference regarding cBDNF concentrations was found between the groups. The serum and CSF BDNF concentrations are shown in Table 4.

**Table 4:** BDNF concentrations in serum and CSF for neurological patients with and without relapse as well as somatoform controls

	sBDNF [pg/ml] mean ± SD	cBDNF [pg/ml] mean ± SD
Neurological patients		
All	28938 ± 10419	0.4167 ± 2.403
With relapse	29301 ± 10494	0.4555 ± 2.656
Without relapse	27348 ± 10145	0.2542 ± 0.6620
Somatoform controls	25407 ± 12696	0.1862 ± 0.5289

No significant differences were found regarding sex, age or kidney function parameters as well as EDSS, severe relapses, poor recovery or lesion count.

### Correlation of different biomarkers in serum and CSF

**Fig. 5:**
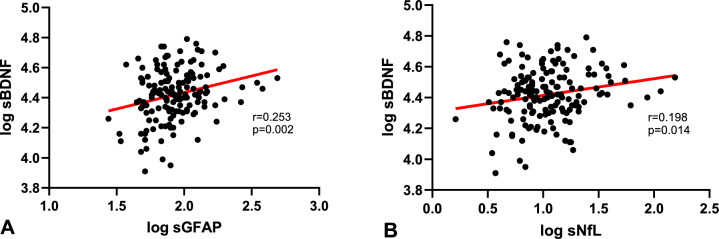
Association of serum BDNF concentration with neuroaxonal and astroglial injury Markers. A significant positive correlation was found for sBDNF with sGFAP (*r* = 0.253, *p* = 0.002, Fig. 4A) as well as with sNfL (*r* = 0.198, *p* = 0.014, Fig. 4B)

sBDNF showed a significant positive correlation with sNfL (*r* = 0.198, *p* = 0.014, Fig. 5B) and sGFAP (*r* = 0.253, *p* = 0.002, Fig. 5A) concentrations while no significant correlations with cNfL and cGFAP were found for cBDNF levels.

## Discussion

In this prospective, single-center study, we found higher sBDNF concentrations in patients with acute inflammatory relapses in IND, such as MS, NMOSD, or MOGAD, compared to somatoform controls while neurological patients without acute relapse did not differ regarding their sBDNF levels compared to somatoform controls.

So far, studies assessing the role of BDNF in pwMS as well as other neurological diseases were inconclusive [26]. While some studies reported decreased BDNF concentrations in pwMS [12–16], other studies found no difference to healthy controls [12, 17–19] or even increased BDNF level [12, 14, 16, 20–23]. However, our findings support the assumption that BDNF might be released in acute inflammatory relapses perhaps as a compensatory mechanism following neuroaxonal damage. This hypothesis is further supported by our findings that sBDNF showed a significant positive correlation with already established biomarkers of neuroaxonal and astroglial injury, such as sNfL and sGFAP. One possible explanation for this weak correlation is the limited cohort size. Alternatively—and this seems rather probable, considering the diagnostic performance differences between sBDNF, sNfL and sGFAP—sBDNF might reflect different mechanisms or steps in neuroinflammation and neuroaxonal damage beyond the ones where sNfL and sGFAP are involved.

BDNF serum level seems to vary with different disease-related characteristics. We found higher concentrations in pwMS with disease onset after the age of 40 years, patients with more than two gadolinium-enhancing lesions and patients with motor, cerebellar, cognitive or sphincter symptoms at disease onset. These clinical and imaging characteristics have been suggested as a hallmark of an aggressive disease course in pwMS [25]. One possible explanation for these findings is that BDNF is elevated as a compensatory mechanism to support remyelination and is therefore more pronounced in patients with severe neuroaxonal and astroglial injury, suggesting that not only inflammatory activity but also remyelination and reserve capacities might vary with disease severity. This is in line with a previous study, where BDNF levels in serum and CSF were higher in patients with significant clinical and cognitive improvement 12 months after acute relapse [28]. Interestingly, we did not find significant differences for sNfL and sGFAP concentrations for these clinicoradiological characteristics though sNfL concentrations have been shown to be increased in patients with at least two gadolinium-enhancing lesions [45]. However, as it is known that these markers of neuroaxonal and glial injury increase in acute relapses as well as progression independent of relapse and correlate with the severity of the neurological deficits [46–48], one possible explanation for our findings could be that BDNF increases are more sensitive in limited cohort sizes or could increase more than NfL or GFAP in some patients or patients with certain clinicoradiological characteristics. Accordingly, BDNF could represent an additional element for evaluation of the prognosis of pwMS at disease onset in addition to NfL and GFAP.

The affinity of receptors to BDNF varies so that a concentration-dependent mechanism could be assumed: While the TrkB receptor has a high affinity for BDNF and exerts neuroprotective effects, the p75-NTR receptor shows a low affinity, thus exerting pro-apoptotic effects at higher BDNF concentrations [5]. Therefore, higher BDNF levels in patients showing characteristics of an aggressive disease course could reflect insufficient repair mechanisms leading to apoptosis and permanent neuroaxonal damage and therefore persisting neurological deficits in patients with MS. Additionally, the truncated isoform of the TrkB receptor, which is the main form on astrocytes, may cause neuronal damage and neurodegeneration via NO production in EAE [49]. However, an association of higher BDNF levels with poor recovery then would be expected, which was not the case in our cohort. Still, as mentioned before, studies already found an association between higher BDNF levels during acute relapses and better improvement from neurological deficits resulting from these relapses [28], thus supporting rather the hypothesis of the potential beneficial role of BDNF in our study.

Additionally, we did not find a significant association between BDNF level and EDSS, severe relapses, or lesion count. This further highlights the importance of carefully assessing the patients’ profile for adequate disease activity predictors. However, regarding EDSS and the classification as severe relapses, a higher interrater variability making these characteristics more vulnerable and thus less objective could be an explanation for the non-significant results. Furthermore, a small lesion resulting from only minimal inflammatory disease activity can cause severe neurological symptoms with a significant EDSS increase, for example when it’s located in the brain stem or the corona radiata. Still, as mentioned before, damaged astrocytes as well as activated microglia were suggested to enhance BDNF expression and secretion. Therefore, higher lesion volume with more damaged astrocytes and more pronounced inflammatory activity would result in higher BDNF concentrations than small lesions. This also counts for lesion count as lesion volume was suggested to reflect the amount of neuroaxonal damage more appropriate [43].

Previous studies have reported an association between BDNF concentration and T2 lesion count, while studies were inconclusive regarding an association with T1 hypointense lesions [38, 39]. However, in context of NfL, it could be shown that lesion volume is more strongly associated with sNfL than lesion number [43] probably due to tumefactive lesions or smoldering lesions as risk factors for progressive disease and long-term disability, which are only reflected in lesion volume, not in lesion count. Accordingly, this also seems conceivable for BDNF against the background of its possible function as a compensatory mechanism. Still, lesion volume was not assessed in the current study, which might explain why no correlation between BDNF concentrations and T2 lesions was found in our study.

By not finding an association between BDNF level and EDSS, our study is in line with some of the previous studies, which also failed to find a relationship between the BDNF concentration, and the disease severity measured as MSSS [27]. However, as studies assessing the association of BDNF and disease severity are scarce and a pilot study showing higher BDNF concentrations being associated with disability improvement after 12 months [28], more studies with larger cohorts and longitudinal study designs are needed to further assess possible associations.

Furthermore, we found no significant differences of BDNF level in neurological patients regarding sex, age or parameters of kidney function.

In contrast to NfL, the impact of renal function and BDNF has mainly been studied in the context of chronic kidney disease [50] with some studies reporting decreased BDNF level in serum in patients with chronic kidney disease compared to healthy controls [51], while other studies reported no change in serum concentrations of BDNF due to impaired renal function as measured by GFR [52]. So far, no studies assessed the impact of kidney function on BDNF serum level in neurological patients. However, as the study did not include any patients with impaired kidney function, the effect of kidney function on BDNF concentrations can only be inferred to a limited extent.

Furthermore, no differences were found regarding cBDNF concentrations. Higher serum concentrations compared to CSF concentrations are thought to result from the peripheral synthesis and release of BDNF by platelets, monocytes and activated T and B cells, which can be triggered by pro-inflammatory cytokines. It has been known for decades that inflammatory mechanisms in the periphery are involved in the pathogenesis of MS [53]. Therefore, pro-inflammatory cytokines, released in the inflammatory milieu of developing MS lesions could enhance the synthesis and release of BDNF in the periphery, thus explaining the association of hallmarks of aggressive MS with sBDNF, but not cBDNF. Still, it is unclear whether BDNF can pass the blood–brain barrier and therefore, whether peripheral synthesized BDNF exhibits neuroprotective effects [26].

### Limitations

Our study is not without limitations. Since it was a monocentric study, only a moderately large sample could be recruited. Furthermore, spinal MRI and some other measurements are not regularly performed in routine clinical practice. Thus, some data such as MRI of the spinal cord were not available in all subjects. However, most of the relevant clinical and imaging parameters could be evaluated for the whole sample. Since both serum and CSF were collected for biomarker analysis in all patients, a more differentiated investigation of BDNF and its levels in neuroinflammation was possible.

Moreover, the study was not designed to analyze recovery from relapses or clinical improvement as no follow up measurement was performed. Hence, a longitudinal assessment of BDNF regarding different clinical characteristics especially disability improvement should be analyzed in future studies with larger cohorts, but also to evaluate the temporal dynamics of BDNF concentrations compared to NfL and GFAP changes as established biomarkers for neuroaxonal and astroglial injury.

## Conclusion

BDNF seems to play a relevant role in neurological diseases, especially in the context of acute inflammatory disease activity (i.e., acute relapse). It is increased when other biomarkers of neuroaxonal damage and neurodegeneration, such as NfL and GFAP, are elevated, possibly as a compensatory mechanism. In pwMS, BDNF serum levels vary depending on important disease-related characteristics, suggesting that not only inflammatory activity but also remyelination and reserve capacities might vary with disease severity. Future studies should focus on the mechanisms underlying these associations and their relevance for predicting the individual disease course.

## Data Availability

The data that support the findings of this study are available from the corresponding author upon reasonable request. The data are not publicly available due to privacy or ethical restrictions.

## Abbreviations

ANOVA: One-way analysis of variance
AUC: Area under the curve
BDNF: Brain-derived neurotrophic factor
ECTRIMS: European Committee for Treatment and Research in Multiple Sclerosis
EDSS: Expanded Disability Status Scale
GAD+: Gadolinium-enhancing lesions
GFAP: Glial fibrillary acidic protein
IND: Inflammatory neurological diseases
IQR: Interquartile range
MOGAD: MOG-antibody associated diseases
MS: Multiple Sclerosis
NfL: Neurofilament light chain
NIND: Non-inflammatory neurological disorders
NMOSD: Neuromyelitis Optica Spectrum disorders
pwMS: Patients with MS
ROC: Receiver operator characteristic
rpm: Runs per minute
